# Primary Schwannoma of the Thyroid Gland in a 71-Year-Old Woman: A Case Report

**DOI:** 10.7759/cureus.36410

**Published:** 2023-03-20

**Authors:** Abdulaziz F Altowairqi, Ahmad S Alharthi, Wahaj A Altalhi, Eidha Aljuaid, Albaraa Y Alsini, Muhammad U Tariq, Areen I Altwairqi

**Affiliations:** 1 Otolaryngology - Head and Neck Surgery, King Abdulaziz Specialist Hospital, Taif, SAU; 2 Otolaryngology - Head and Neck Surgery, Alhada Armed Forces Hospital, Taif, SAU; 3 Medicine, Taif University, Taif, SAU; 4 Head and Neck Surgery, Alhada Military Hospital, Taif, SAU; 5 Otolaryngology - Head and Neck Surgery, Alhada Military Hospital, Taif, SAU; 6 Histopathology, Alhada Armed Forces Hospital, Taif, SAU; 7 Medicine, King Saud bin Abdulaziz University for Health Sciences, Jeddah, SAU

**Keywords:** neoplasm, nerve, gland, thyroid, schwannoma

## Abstract

Schwann cells in the body's nerve sheath can develop into benign tumors known as schwannomas. While thyroid gland schwannomas are uncommon and are rarely documented in the literature, they are less unusual than those appearing in the head and neck region. The rare nature of schwannomas connected to the thyroid gland adds to the difficulty in detecting them prior to surgery. At present, the most popular form of treatment for thyroid schwannomas is surgical resection, which is considered to be curative. A mass excision or lobectomy has a favorable prognosis, few postoperative complications, and a low risk of tumor recurrence. This paper reports the case of a 71-year-old woman who presented with left neck swelling that had been increasing in size over a number of years. An ultrasonography examination revealed multiple bilateral thyroid nodules with high vascularity. The patient’s right thyroid lobe exhibited benign nodular hyperplasia while the thyroid tissue of the isthmus exhibited benign nodular hyperplasia and schwannoma. Following the diagnosis, the patient's mass was successfully surgically removed.

## Introduction

Schwannomas and neurofibromas are types of nerve sheath tumors that, while histologically distinct, both originate from the Schwann cells surrounding the nerve roots. Schwannomas are benign, slow-growing tumors that can develop from nerve roots anywhere in the body and are often referred to as neuromas, neurinomas, or neurilemomas. Between 25% and 45% of malignant cancers originate in the head or neck. The most frequently affected areas are the eighth cranial nerve, the fifth and seventh cranial nerves, the lateral cervical region, and the mouth cavity [1]. While primary schwannomas in other head and neck sites have been recorded, very few cases of schwannomas at these sites are reported in the literature [2].

 It can also prove difficult to distinguish schwannomas from thyroid nodules due to the similarity of their sonographic characteristics and clinical symptoms [2]. The vestibulocochlear nerve (CN VIII), trigeminal nerve (CN V), and facial nerve (CN VII) are the most common sources of benign schwannomas [3] while primary schwannomas have occasionally developed in the thyroid gland, with the first occurrence reported in 1964 [4].

Uri et al. reported that a 57-year-old woman with Hashimoto's thyroiditis was examined for hypothyroidism. A single large nodule that was cold on a technetium-99m (99mTc) thyroid scan was found to be visible on a thyroid ultrasound scan. Following fine needle aspiration (FNA), the nodule was suspected to be a malignant thyroid tumor. The subsequent thyroidectomy was performed in its entirety without incident. A diagnosis of primary thyroid schwannoma was supported by microscopic analysis and immunohistochemistry staining [3].

Abbarah et al. reported the case of a 33-year-old woman with a rare form of thyroid schwannoma who presented with swelling on the left side of her neck, which induced a change in voice and led to trouble in swallowing [5]. In contrast to the typical primarily solid nodule described in the literature, an ultrasound examination of the mass revealed a huge heterogeneous, predominantly cystic lesion. Despite extensive preoperative diagnostic testing, which included FNA, the diagnosis remained unclear. However, the mass was ultimately successfully removed surgically, and schwannoma was diagnosed based on the histopathological analysis, which indicated the presence of type A and type B Antoni cells and positive staining for S-100 protein.

Herein, a case of an older woman with primary thyroid schwannoma is presented. The patient provided written informed consent, and the institutional review board gave its approval for the case to be published.

## Case presentation

This case concerns a 71-year-old female, known case of diabetes mellitus and hypertension, referred from endocrinology as a case of primary hyperparathyroidism. She was complaining of generalized body and bone aches for around two years. There was no history of kidney stones or fractures during this period. There were no other ENT symptoms. The systemic review was unremarkable. On examination, the patient appeared well, with normal vital signs. There was no palpable neck swelling or lymphadenopathy, and the fiberoptic laryngoscopy indicated bilateral mobile vocal cords. The laboratory tests revealed a high parathyroid hormone level (441 pg/mL), high calcium level (28 mg/dL), and normal thyroid function test.

An ultrasound examination revealed multiple bilateral thyroid nodules with high vascularity. The right lobe measured 5 × 1.5 × 1.5 cm, indicating increased vascularity and a large lower lobe hyperechoic heterogeneous nodule measuring 1.5 × 1.5 cm. The left lobe measured 5.3 × 2 × 1.7 cm and exhibited multiple hypoechoic solid, well-defined nodules. The largest nodule at the upper mid-lobe measured 2.5 × 1 cm, which indicated internal vascularity.

A 99mTc-sestamibi evaluation indicated bilateral inferior parathyroid adenoma while the combined 99mTc-pertechnetate and 99mTc-methoxyisobutylisonitrile (MIBI) single-photon emission computed tomography parathyroid imaging revealed a multinodular goiter. Early and late myocardial perfusion imaging (MIBI) revealed two areas of retained activity below both thyroid lobes (two inferior parathyroid adenomas).

The patient underwent left inferior parathyroid adenoma excision and right hemithyroidectomy. The intraoperative dropping of parathyroid hormone by more than 50% from 48 pg/mL to 6 pg/mL, with no complications, was encountered intraoperatively, and the postoperative course was uneventful.

**Figure 1:**
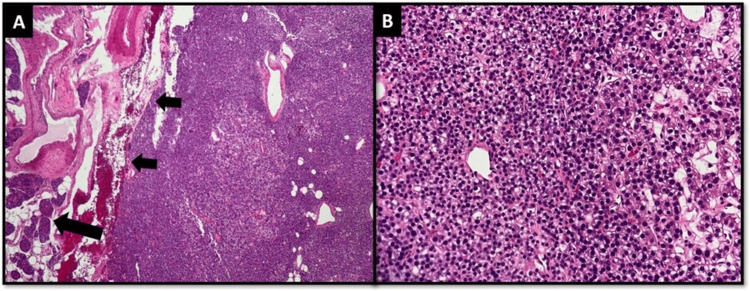
Microscopic examination of the parathyroid gland tissue A) Parathyroid tumor surrounded by a thin fibrous capsule (small arrows); normal parathyroid gland tissue was also identified at the periphery (larger arrow); B) The tumor was predominantly composed of sheets of chief cells along with a few clear cells

The macroscopical features included the right inferior parathyroid gland measuring 0.5 × 0.5 × 0.4 cm, the left inferior parathyroid gland measuring 2 × 1 × 0.8 cm, and the right thyroid lobe measuring 3.5 × 2.1 × 2 cm. No solid area was identified. The specimen labeled as “isthmus” was thyroid tissue with an attached nodular encapsulated lesion measuring 2.6 × 1.6 × 1 cm. The histological features and microscopic examination of the right and left inferior parathyroid glands revealed circumscribed lesions surrounded by a thin fibrous capsule. A normal parathyroid gland was also observed outside of the capsule (Figures 1A, 1B). Finally, a diagnosis of parathyroid adenoma was reached.

**Figure 2:**
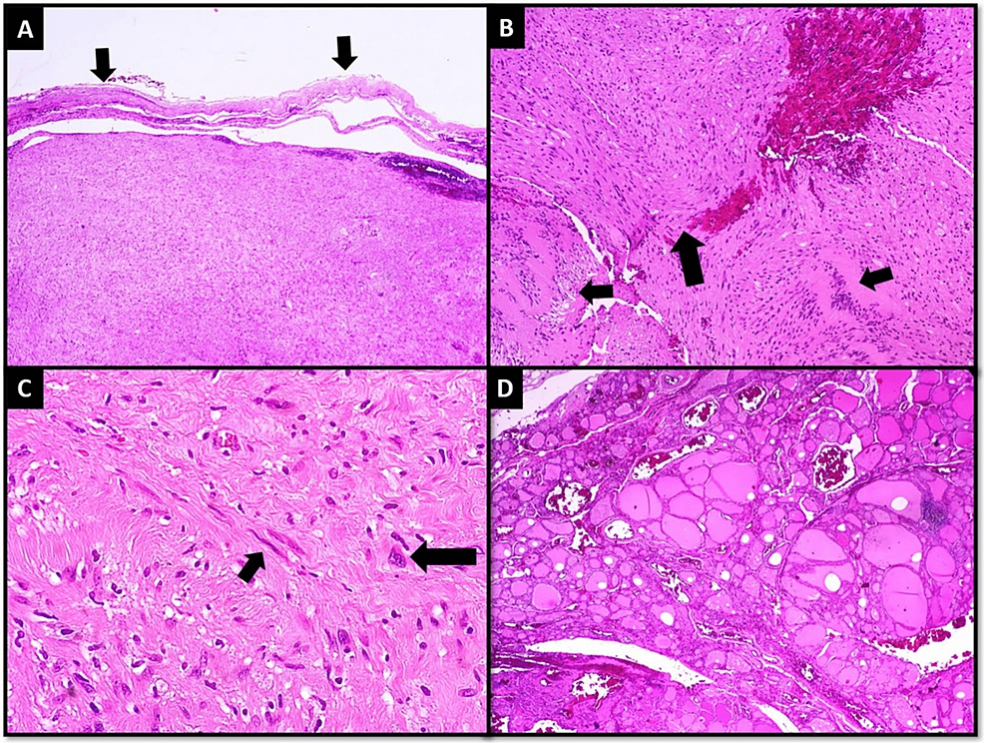
Microscopic examination of right thyroid lobe and isthmus A) Nodular lesion exhibiting a spindle cell lesion surrounded by a thin capsule (arrows); B) Tumor cells exhibiting nuclear palisading (small arrows) and Verocay bodies (larger arrow); C) Tumor cells exhibiting elongated, wavy nuclei with tapered ends (smaller arrow) and degenerative atypia (larger arrow); D) Right thyroid lobe exhibiting benign nodular hyperplasia

The microscopic examination of the nodular lesion labeled as “isthmus” revealed an encapsulated benign spindle cell lesion arranged in fascicles of spindle cells exhibiting nuclear palisading and associated Verocay bodies. A number of the neoplastic cells exhibited elongated, wavy nuclei with tapered ends, while degenerative atypia was also observed in certain cells. Increased mitoses, nuclear pleomorphism, and necrosis were not observed. Based on the histological features, a diagnosis of schwannoma was reached. The right thyroid lobe and thyroid tissue labeled “isthmus” exhibited benign nodular hyperplasia (Figures 2A-2D).

## Discussion

Schwannomas are slow-growing, encapsulated tumors that develop from Schwann cells and other neural sheath cells. Although schwannomas frequently affect the head and neck, it is uncommonly involved in the thyroid gland [2]. The intrathyroidal sensory nerves or thyroid autonomic innervation are assumed to be the source of primary thyroid schwannomas [6].

Our patient presented with a painless neck swelling that was increasing in size. In this case, the 99mTc examinations revealed two inferior parathyroid adenomas. Thyroid adenomas, which are benign epithelial tumors, account for around 80% of nonfunctioning thyroid nodules (cold nodules), with the remaining 20% being malignant. Every suspicious nodule must undergo a cytologic investigation due to the increased risk of cancer in cold thyroid nodules, which, in fact, occurred in this case. Thyroid non-epithelial tumors are highly uncommon, with extremely few instances reported in the literature [7,8].

The sonographic findings revealed multiple bilateral thyroid nodules with high vascularity. Alsop et al. reported that thyroid-related schwannomas are uncommon and challenging to identify prior to surgery [9]. They frequently manifest as a painless neck mass, and it can be challenging to differentiate them from most thyroid nodules on ultrasound examination. In fact, a thyroid schwannoma is rarely definitively identified prior to a thyroid lobectomy, with the diagnosis occasionally emerging by chance via a pathology examination following surgical intervention.

Our patient underwent left inferior parathyroid adenoma excision and right hemithyroidectomy. The right thyroid lobe exhibited benign nodular hyperplasia while the thyroid tissue of the isthmus exhibited benign nodular hyperplasia and schwannoma. The specimen from the isthmus consisted of thyroid tissue and an attached nodular lesion.

Depending on the Antoni cell structure and cystic alterations, schwannomas can display variable degrees of echogenicity on ultrasound examination, while, in general, they do so to a lesser extent than muscle tissue [10,11]. Since calcification in schwannomas is so uncommon, it can prove challenging to develop a differential diagnosis. In fact, only a few papers have discussed instances of calcification in schwannomas, even though it is a common finding associated with degenerative alterations [12,13].

In our patient, the tumor's marked hypoechogenicity and the appearance of echogenic foci that indicated macro- and micro-calcifications complicated the differential diagnosis and raised suspicion of malignancy [14].

To summarize, primary thyroid schwannomas are extremely uncommon, and the few cases described in the literature vary in terms of age group, presented symptoms, clinical course, and histological appearance. Complete surgical excision is deemed to be curative and, thus far, would appear to be the preferred course of action.

In our case, the diagnosis of primary thyroid schwannoma was an Incidental finding in the histopathology report post-right hemithyroidectomy. A literature review of previously reported cases is summarized in Table 1.

**Table 1:** Summary of previously reported cases of thyroid schwannoma

case	Age\sex	presentation	US finding	FNA result	Surgery	Definitive pathology
Ledgard C et al., 2022 [15]	33/F	Large palpable mass, compression.	53 × 19 × 19 mm—Inferior left thyroid nodule, Solid and hypoechoic with minimal internal vascularity (TIRADS 4)	Non- diagnostic (acellular).	Nodule removal.	Encapsulated mass (schwannoma) arising adjacent to nerve bundles. Spindle cells with both Type A and B Antoni cells.
Kang et al., 2019 [16]	33/F	None.	30 mm—Inferior left thyroid nodule, Well-defined, oval-shaped, markedly hypoechoic intrathyroidal nodule with echogenic foci and macro- and microcalcifications.	Non- diagnostic.	Left hemi- thyroidectomy.	Schwannoma in the perithyroid tissue, with compact areas of spindle cells (Antoni A) and loosely arranged foci (Antoni B).
Nagavalli et al., 2017 [17]	60/F	Dysphagia, neck mass.	Left thyroid lobe, Ovoid hypoechoic mass with smooth borders.	Spindle cells with a lymphocytic background.	Nodule removal.	Spindle cells in whorls with a predominantly Antoni A pattern.
Donatini et al., 2008 [18]	26/F	Neck mass (Thyroid cancer with cervical- lymph node metastases).	38 mm—Right thyroid lobe, nodular lesion close to the neck vascular bundle suspicious for a metastatic lymph node.	Fibrous tissue without any tumoral cells.	Mass removal.	Schwannoma.
Cashman et al., 2008 [6]	35/F	Neck mass; right Horner’s syndrome.	6 × 2.4 × 2.7 mm—Right thyroid lobe, Large mass arising from the thyroid gland.	Inconclusive.	Right hemi- thyroidectomy.	Schwannoma with spindle cells without atypia, mitosis, or necrosis.
De Paoli et al., 2005 [10]	63/F	Foreign body sensation with swallowing.	27 mm—Lower pole of the right thyroid lobe, Markedly hypoechogenic nodule with rich vascularity.	Fragments of adipose tissue, rare thymocytes in aggregates resembling follicular masses, insufficient for diagnosis.	Total thyroidectomy.	Schwannoma in the perithyroid tissue, with compact areas of spindle cells or Verocay bodies (Antoni A) and loosely arranged foci (Antoni B).

## Conclusions

In this report, the case of an older woman with a right thyroid lobe that exhibited benign nodular hyperplasia was presented. The thyroid tissue of the isthmus exhibited benign nodular hyperplasia and schwannoma. Thyroid tissue and a nodular lesion were both present in the isthmus samples.

Thyroid-related schwannomas are rare and challenging to identify prior to surgery. In terms of sonographic appearance, this type of tumor closely resembles a thyroid nodule. Surgical resection is the most common treatment for thyroid schwannoma and is considered curative. A mass excision or lobectomy typically has a good prognosis, few postoperative complications, and little chance of tumor recurrence. In this case, the mass was successfully removed surgically.
